# A C2H2 Zinc Finger Protein FEMU2 Is Required for *fox1* Expression in *Chlamydomonas reinhardtii*

**DOI:** 10.1371/journal.pone.0112977

**Published:** 2014-12-08

**Authors:** Xiaodong Deng, Jinghao Yang, Xiaoxia Wu, YaJun Li, Xiaowen Fei

**Affiliations:** 1 Key Laboratory of Biology and Genetic Resources of Tropical Crops, Ministry of Agriculture, Institute of Tropical Bioscience and Biotechnology, Chinese Academy of Tropical Agricultural Science, Haikou, 571101, China; 2 School of Science, Hainan Medical College, Haikou, 571101, China; University of Florida, United States of America

## Abstract

*Chlamydomonas reinhardtii fox1* gene encodes a ferroxidase that is involved in cellular Fe uptake and highly induced during Fe deficient conditions. In an effort to identify *fox1* promoter regulatory elements, an insertional library was generated in a transgenic *Chlamydomonas* strain (2A38) harboring an arylsulfatase (ARS) reporter gene driven by the *fox1* promoter. Mutants with a defective response to low iron conditions were selected for further study. Among these, a strain containing a disrupted *femu2* gene was identified. Activation of the *fox1* promoter by the *femu2* gene product was confirmed by silencing the *femu2* gene using RNA interference. In three *femu2* RNAi transgenic lines (IR3, IR6, and IR7), ARS reporter gene activities declined by 84.3%, 86.4%, and 88.8%, respectively under Fe deficient conditions. Furthermore, *RT*-PCR analysis of both the *femu2* mutant and the RNAi transgenic lines showed significantly decreased transcript abundance of the endogenous *fox1* gene under Fe deficient conditions. Amino acid sequence analysis of the *femu2* gene product identified three potential C2H2 zinc finger (ZF) motifs and a nuclear localization study suggests that FEMU2 is localized to the nucleus. In addition, a potential FEMU2 binding site ((G/T)TTGG(G/T)(G/T)T) was identified using PCR-mediated random binding site selection. Taken together, this evidence suggests that FEMU2 is involved in up-regulation of the *fox1* gene in Fe deficient cells.

## Introduction

C2H2 zinc finger proteins (ZFPs) comprise an abundant family of nucleic acid-binding proteins in eukaryotic genomes. The number of ZFPs identified from in silico analyses corresponds to approximately 2.3% and 3% of the genes in Diptera and Mammal families, respectively [1], [2]. Approximately 0.8% and 0.7% of *Saccharomyces cerevisiae* and *Arabidopsis thaliana* proteins have C2H2 zinc finger (ZF) domains [3], [4]. C2H2 ZFs have numerous functions, ranging from DNA and RNA binding to protein–protein interactions. C2H2 ZFPs are reportedly involved in cell or tissue development, stress response, and other regulatory processes in organisms [5]–[9].

Many stress-responsive C2H2 ZFPs have been identified in various plant species. Studies have reported that C2H2 ZFP gene overexpression activates stress-related genes and enhances salt tolerance, dehydration, and cold stress [10]–[13].

In the photosynthetic eukaryote model *Chlamydomonas reinhardtii*, iron (Fe)-deficiency causes a series of cellular reactions. Among these reactions, a high-affinity system that consists of iron reductase (FRE1), a protein involved in iron assimilation (FEA1), and a transport complex comprising a multi-copper oxidase (FOX1) and an Fe transporter (FTR1), are induced [14], [15]. Fe^3+^ is reduced by FRE1 to Fe^2+^, which is mediated intracellularly by FEA1 and subsequently re-oxidized to Fe^3+^ by FOX1 at the FTR1 site. This process transports Fe into cells. Expressions of *fox1, ftr1, fre1, fea1*, and antioxidant 1 (*atx1*) have been linked to Fe deficiency [16]–[22]. Other genes involved in the regulation of Fe and other metal ions have been identified by genomic analyses, which belong to several gene families including CDF (*mtp1* to *mtp*5), ZIP (*zip1* to *zip14*), COPT (*copt1*), and NRAMP (*nramp1* to *nramp3*) families [23], [24]. However, information on the genes regulating Fe deficiency-related stress remains insufficient. Thus far, no study has been reported on C2H2 ZFPs in *Chlamydomonas*.

In previous studies, *fox1* FeRE1 (Fe reaction element) at −87/−82 (CACACG) and FeRE2 at −65/−61 (CACGCG) were identified. These elements are essential in inducing *fox1* expression under Fe-deficient conditions. The results from scanning mutagenesis analyses revealed a *fox1* FeRE consensus sequence C(A/G)C(A/G)C(G/T) [25]. Subsequently, a plasmid pARG7.8 used in the selection of transformants was cotransformed with the plasmid pJF103, which contained the *fox1* promoter sequence (−103 to +65) and arylsulfatase (ARS) reporter gene, to *C. reinhardtii* CC425(cw15 arg2). A transformant named 2A38 with relative high ARS activity (5.7 nmol p-nitrophenol min^−1^ ×10^−6^ cells) in iron-free medium were obtained using a selective arginine-deficient tris-acetate phosphate (TAP) medium. After the ARS substrate 5-bromo-4-chloro-3-indolyl sulfate (XSO4) was added, the 2A38 clones appeared deep blue in the Fe-free TAP solid medium. Thus, 2A38 was used as the recipient strain in insertion mutation. To obtain insertion mutants, zeocin-resistant pSP124S plasmid was used to transform *C. reinhardtii* 2A38, which containing *fox1* promoter::*ars* chimeric gene, in our study. The transformants were divided in two groups by ARS phenotype analysis, e.i., effective mutants and ineffective mutants. After the ARS substrate XSO4 was added, effective mutants appeared white or in different hues of light blue comparing with the deep blue of 2A38 phenotype. Ineffective mutants showed similar deep blue color as the2A38 parent strain. A total of 68 effective mutants were identified in approximately 50,000 zeocin-resistant transformants. Wherein, the Fe-deficiency response-defective mutant mu2 has been further studied in this work.

## Results

### mu2 isolation and analysis

**Figure 1:**
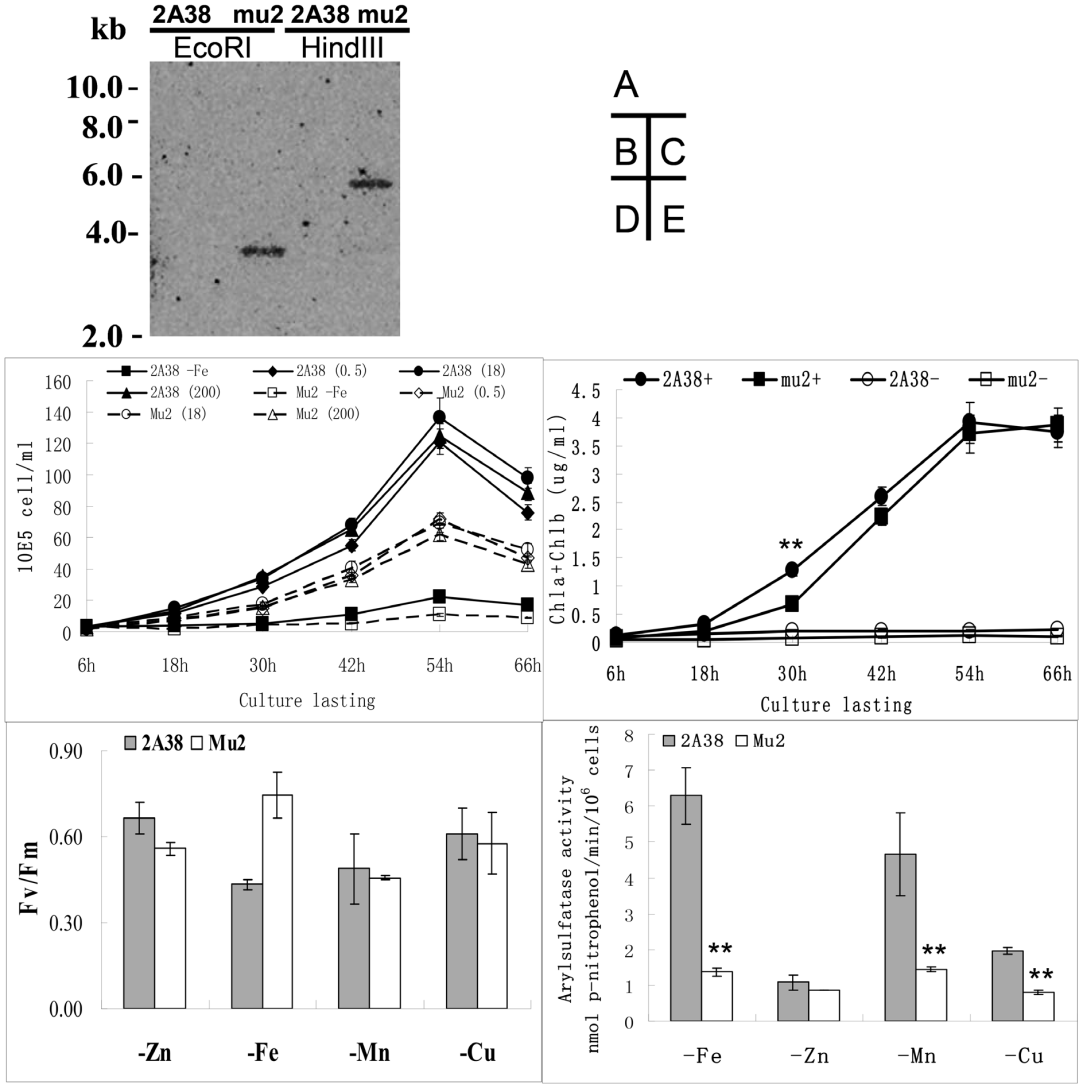
Analysis of *C. reinhardtii* mu2. (A) Analysis of DNA insertion in *C. reinhardtii* mu2 by DNA gel blot. After the total DNA of *C. reinhardtii* mu2 and *C. reinhardtii* 2A38 were digested with *Eco*RI or *Hind*III and verified using agarose gel electrophoresis and gel blot analysis, the mutant gene was hybridized with a 100-bp P^32^-labeled *ble* CDS DNA fragment. (B) The growth curve of *C. reinhardtii* mu2 and parent strain *C. reinhardtii* 2A38. -Fe indicates the algae was cultured in 0 µM Fe TAP medium; 0.5, 18, and 200 mean that the algae were cultured in 0.5, 18, and 200 µM Fe TAP medium, respectively. (C) The chlorophyll content of *C. reinhardtii* mu2 and parent strain *C. reinhardtii* 2A38. + indicates that the algae was cultured in 18 µM Fe TAP medium; - indicates that the algae was cultured in 0 µM Fe TAP medium. (D) Primarily photochemical efficiency of photosystem II (Fv/Fm) assay of *C. reinhardtii* mu2 and parent strain *C. reinhardtii* 2A38 cultivated in –Zn, –Fe, –Mn, and –Cu TAP medium after 4 days. (E) Quantity analysis of ARS activity of *C. reinhardtii* mu2 and parent strain *C. reinhardtii* 2A38 cultivated in –Zn, –Fe, –Mn, and –Cu TAP medium after 4 days. T-test was conducted on the experiments using SPSS statistical software. Significance was set to *P<0.05, ** *P*<0.01.

To identify the *C. reinhardtii* genes involved in cellular responses to Fe deficiency, *C. reinhardtii* 2A38 was subjected to random insertion mutagenesis, in which an expression cassette with −103 to +65 *fox1* promoter sequence and an ARS reporter gene was integrated into the genome [25]. Cells from 2A38 were transformed using pSP124S, which contains the *ble* gene that expresses resistance to zeocin (bleomycin). Zeocin-resistant transformants were then transferred onto –Fe plates with 300 µL 10 mM XSO_4_ to determine ARS reporter gene activity. Given that the recipient cell *C. reinhardtii* 2A38 contained the *fox1* promoter::*ars* expression cassette, the transformants were likely to show no blue halo around their colonies if the insertion affected the genes controlling the *fox1* gene transcription or those participating in signal transduction in response to Fe deficiency. This approach was used to isolate the mutant strain mu2, which contains a single *ble* insertion locus that is integrated into its nuclear genome (Fig. 1A). The mutant mu2 grew significantly slower than the parent strain 2A38 in the +Fe medium (0.5, 18, and 200 µM Fe). However, no obvious growth differences were observed between the mutant and the parent strain in 0 µM –Fe medium (Fig. 1B). Moreover, no significant difference in chlorophyll content was observed between the mutant and the parent strain in both +/– Fe TAP media (Fig. 1C). Primarily photochemical efficiency of photosystem II (Fv/Fm) in mu2 increased in –Fe conditions compare to in parent strain, but no significant difference was observed between the two in –Zn, –Fe, –Mn, and –Cu TAP media, indicating that mutation did not affect the photosynthesis of mu2 (Fig. 1D). ARS activity of mu2 was significantly lower than that of 2A38 in the –Fe, –Mn, and –Cu TAP media, but no significant difference was found between the two in the –Zn TAP medium (Fig. 1E). Given the crosstalk among the various nutrient deficiencies, the results in –Mn and –Cu conditions could have been caused by secondary Fe deficiency and were not a direct effect of Mn or Cu.

### Identification of the gene responsible for ARS activity in mu2

**Figure 2:**
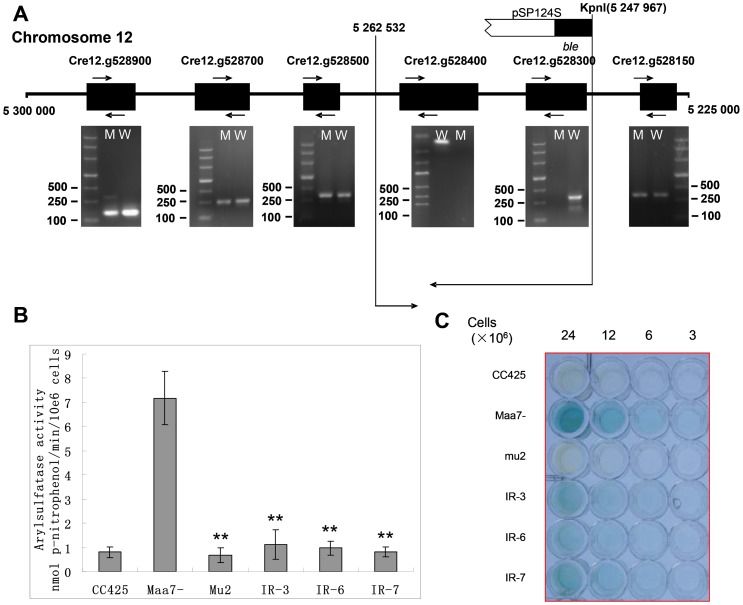
Identification of the gene responsible for ARS activity in mu2. (A) Schematic diagram of pSP124S insertion into the *C. reinhardtii* mu2 genome. pSP124S was inserted into the *C. reinhardtii* mu2 at the scaffold 31 149712 site (JGI, Chlre 3), with about 1.4 kb of the DNA fragment deleted (from 135000 to 149712). Genes 150664 and 192547 were included in this region. M stands for *C. reinhardtii* mu2; W stands for *C. reinhardtii* 2A38. The protein ID number denotes the corresponding genes. (B) Comparison of ARS activity of transgenic *C. reinhardtii*. (C) ARS activity analysis using XSO4 as substrate. CC425, non-transgenic strain without *fox1* promoter::*ARS* expression cassette; Maa7-, transgenic algae with pMaa7IR/XIR transformed into *C. reinhardtii* 2A38; mu2, mutant with pSP124S inserted into the *C. reinhardtii* 2A38 genome; and IR3-IR7, pMaa7IR/Femu2IR transgenic algae. ANOVA (combined with Duncan's Multiple Range Test) was conducted on the experiments using SPSS statistical software. Significance was set to *P<0.05, ** *P*<0.01.

The DNA sequence flanking one end of the introduced *ble* gene was determined by thermal asymmetric interlaced-PCR (TAIL-PCR). To determine the length of the DNA sequence disrupted by mutagenesis, a series of primers around the *ble* insertion site between Cre12.g528300 and Cre12.g528150 genes (Table 1) was designed using the parent strain and mutant DNA as PCR templates (Fig. 2A). The results showed that the target band could be amplified using the DNA template from the parent strain and mu2 in Cre12.g528900, Cre12.g528700, Cre12.g528500, and Cre12.g528150 genes. For Cre12.g528400 and Cre12.g528300 genes, the target band was only amplified using the DNA template from the parent strain. This result indicated that the mutant DNA between Cre12.g528400 and Cre12.g528300 genes was deleted by insertion mutagenesis. Thus, Cre12.g528400 and Cre12.g528300 genes could respond to induced Fe deficiency in *Chlamydomonas.*

**Table 1:** Tail-PCR cycling parameters used to isolate flanking DNA from inspectional mutants.

Reaction	Step	Thermal settings	No. of cycles
Primary	1	95°C, 2 min	1
	2	94°C, 1 min; 62°C, 1 min; 72°C, 2.5 min	5
	3	94°C, 1 min; 25°C, 3 min; ramping to 72°C over 3 min; 72°C, 2.5 min	1
	4	94°C, 30 s; 68°C, 1 min; 72°C, 2.5 min; 94°C, 30 s; 68°C, 1 min; 72°C, 2.5 min; 94°C, 30 s; 44°C, 1 min; 72°C, 2.5 min	15
	5	72°C, 5 min	1
Secondary	1	94°C, 30 s; 64°C, 1 min; 72°C, 2.5 min; 94°C, 30 s; 64°C, 1 min; 72°C, 2.5 min; 94°C, 30 s; 44°C, 1 min; 72°C, 2.5 min	12
	2	72°C, 5 min	1
Tertiary	1	94°C, 30 s; 44°C, 1 min; 72°C, 1 min;	20
	2	72°C, 5 min	1

### Femu2 silencing led to loss of ARS activity in *C. reinhardtii* 2A38 under Fe-deficient conditions

**Figure 3:**
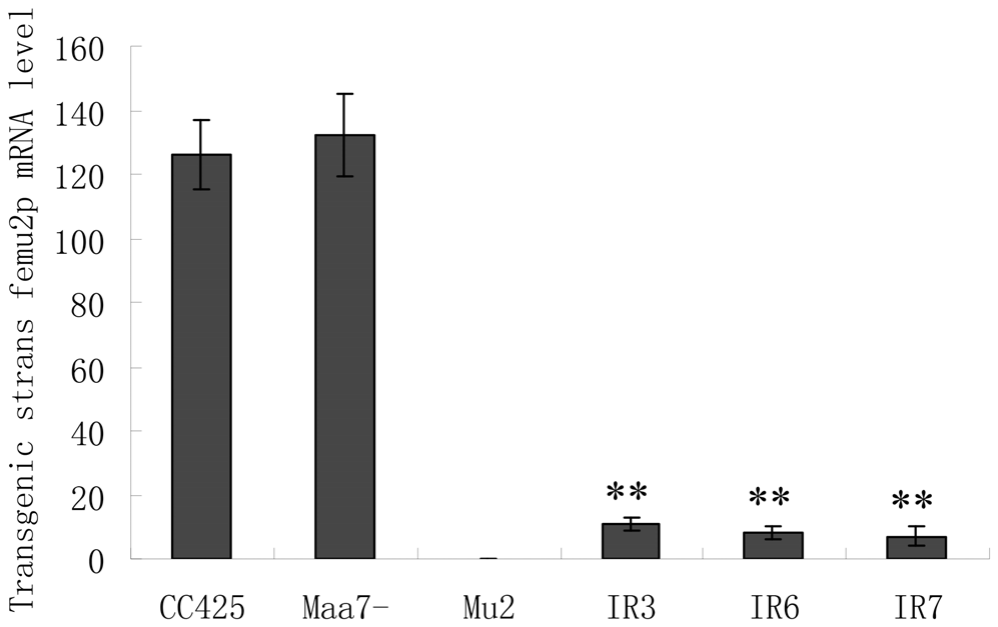
Comparison of mRNA abundance of *femu2* in transgenic *C. reinhardtii*. CC425, non-transgenic strain without the *fox1* promoter::*ars* expression cassette; Maa7-, transgenic algae with pMaa7IR/XIR transformed into *C. reinhardtii* 2A38; mu2, mutant with *femu2* gene deleted from the *C. reinhardtii* 2A38 genome; IR3-IR7, pMaa7IR/Femu2IR transgenic algae; ANOVA (combined with Duncan's Multiple Range Test) was conducted on the experiments using SPSS statistical software. Significance was set to *P<0.05, ** *P*<0.01.

Cre12.g528400 and Cre12.g528300 genes were artificially silenced by using RNAi to determine their effects on ARS activity under Fe-free conditions in *C. reinhardtii*. To amplify the fragments of the Cre12.g528400 coding region, the following primers were designed on the basis of the sequences of Cre12.g528400 gene from the JGI *C. reinhardtii* v4.0 database: 150664F (CCTTCAACGCAGAGGACTTC) and 150664R (TGAATGCAGCCATACTGGAA). This DNA fragment was subcloned and then used to generate the Cre12.g528400 RNAi construct pMaa7IR/*150664* IR. More than 120 transformants carrying the Cre12.g528400 RNAi construct were generated, and three transgenic lines (IR3, IR6 and IR7) were selected for further test. The ARS activity and Cre12.g528400 mRNA abundance under –Fe condition were determined in these transgenic lines, and the non-transgenic *C. reinhardtii CC425* strain and a *C. reinhardtii* 2A38 strain transformed with the pMaa7IR/XIR vector were used as the negative and positive controls respectively. ARS activity decreased by 84.3% (IR3), 86.4% (IR6), and 88.8% (IR7) in cells harboring silenced Cre12.g528400 constructs compared to the positive control line (Figs. 2B and 2C). To evaluate the effectiveness of the RNAi constructs, the mRNA abundance of Cre12.g528400 gene was analyzed using real-time PCR. mRNA abundance in IR3, IR6, and IR7 transgenic strains decreased by 91.7%, 93.9%, and 94.5%, respectively (Fig. 3), indicating high efficient of of Cre12.g528400 RNAi. These results demonstrates that the Cre12.g528400 gene knockdown in *C. reinhardtii* 2A38 suppressed most of the ARS activity, consistent with the loss of ARS activity in *C. reinhardtii* 2A38 knockout mutant mu2. As *C. reinhardtii* 2A38 genomic DNA contains the *fox1* promoter::*ars* expression cassette, both strains of 2A38 and pMaa7IR/XIR transgenic algae retained their ARS activities under –Fe conditions. Gene mutation and RNAi silencing of Cre12.g528400 suppressed the expression of *fox1* promoter::*ars* chimeric gene, indicating that Cre12.g528400 was involved in the regulation of *fox1* promoter::*ars* chimeric gene expression. In contrast, *C. reinhardtii* 2A38 transformed with Cre12.g528300 RNAi did not show suppressed ARS activity. Thus, Cre12.g528400, but not Cre12.g528300 regulated *fox1* promoter::*ars* chimeric gene expression and was named as *femu2* (Fe deficiency mutant 2).

### FEMU2 contains three C2H2 ZF elements for DNA binding

**Figure 4:**
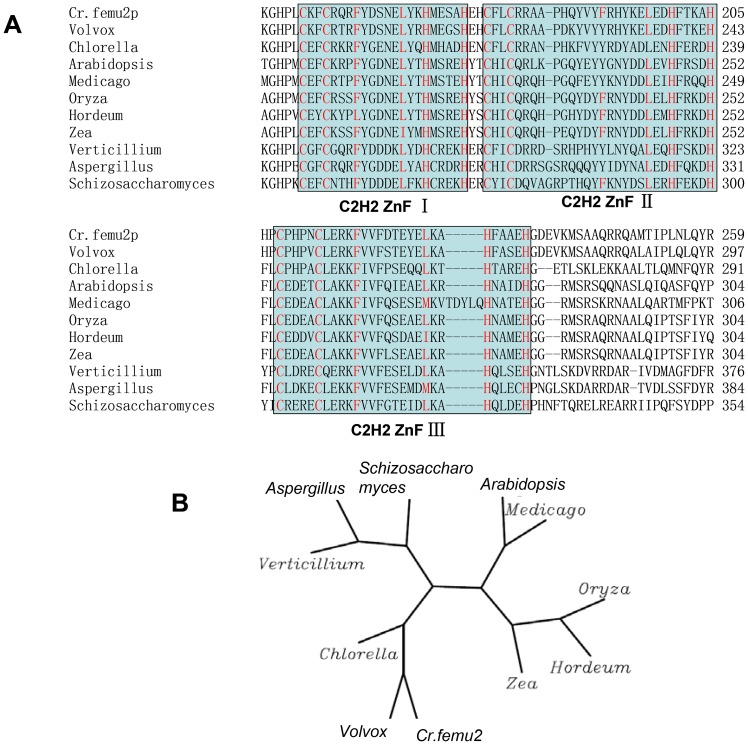
Bioinformatics analysis of FEMU2. (A) Amino acid sequence alignment of FEMU2 3C2H2 domain with its homologous proteins in algae, fungi, and plant. Red letters indicate the conserved amino acid of the C2H2 ZF. Three of the C2H2 domains predicted by Pfame and SMART are indicated by frame. (B) Phylogenetic analysis of *C. reinhardtii* FEMU2 with its homologous genes in other species. Cr.Femu2: *C. reinhardtii femu2* gene (150664); *Volvox, Volvox carteri* XP_002953405; *Chlorella, Chlorella variabilis* EFN57887; *Oryza, Oryza sativa* NP_001055084; *Zea, Zea mays* NP_001169385; *Hordeum, Hordeum vulgare* BAJ93864; *Arabidopsis, Arabidopsis thaliana* NP_566094; *Medicago Medicago, truncatula* XP_003626029; *Schizosaccharomyces, Schizosaccharomyces pombe* NP_588346; *Verticillium, Verticillium dahliae* EGY21451; *Aspergillus, Aspergillus flavus* XP_002373060.

**Figure 5:**
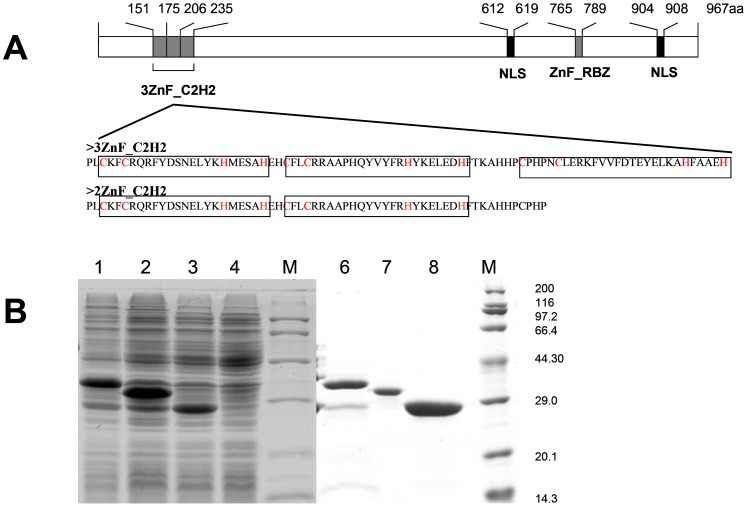
Schematic diagram of FEMU2 C2H2 ZF domain and expression of FEMU2 C2H2 ZF domain. (A) Schematic diagram of the function domains of FEMU2 protein; (B) Expression and purification of FEMU2 3 of C2H2 ZF domain and 2 of the C2H2 ZF domain in *E. coli* BL21 after induced by IPTG. M, Marker; lane1, GST-3C2H2 fusion protein (35.4 KD); 2, GST-2C2H2 fusion protein (32.5 KD); 3, GST only (26 KD); 4, *E. coli* BL21 total protein; 6, purified GST-3C2H2 fusion protein; 7, purified GST-2C2H2 fusion protein; 8, purified GST.

**Figure 6:**
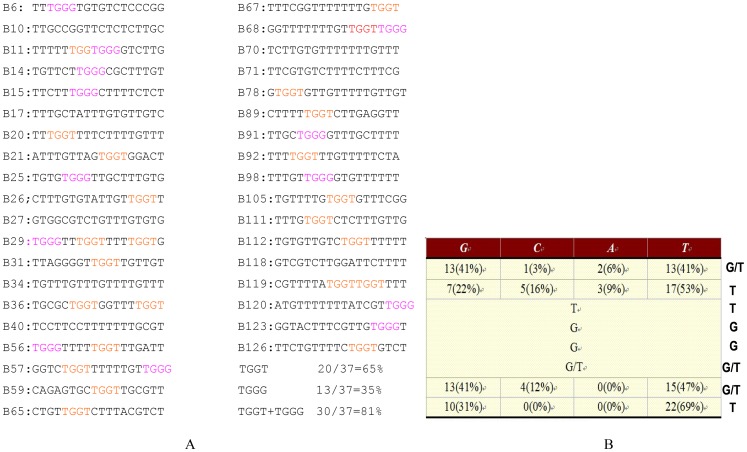
The random DNA sequences binding to FEMU2 3C2H2 ZF. (A) List of the 37 of random binding oligonucleotide sequences selected by FEMU2 3C2H2 ZF. Among them, 20 contain the TGGT core sequence, accounting for 65% of the total sequence; 13 contain the TGGG core sequence, accounting for 35% of the total sequence; and 30 contain TGGG/T the core sequence, accounting for 81% of the total sequences. (B) Frequency of flanking nucleotide selection. The consensus-binding sequences (G/T)TTGG(G/T)(G/T)T are also given.

*Femu2* gene (3904 bp based on the putative gene model) contains seven introns and eight exons. Its 2901-bp CDS encoded 967 amino acids with three typical C2H2 ZF domains (amino acids 151 to 174, 175 to 205, and 206 to 235), as predicted by Pfam and the Simple Modular Architecture Research Tool (SMART; http://smart.embl-heidelberg.de/; Fig. 4A). However, no plant-specific QALGGH sequence was found. FEMU2 is predicted to be localized in the nucleus according to Euk-mPLoc 2.0 software. Moreover, two nuclear localization sites (NLSs; 612-KGKGRAKV-619 and 904-KGKKG-908) were found using NLStradamus (Fig. 5A). FEMU2 homologs was identified by BLAST search from NCBI database, and cluster analysis showed that *Chlamydomonas* FEMU2 is clustered with its homologs in *Volvox* and *Chlorella*. FEMU2 was closest to its homologous proteins in fungi, followed by those in rice, maize, barley, and *Arabidopsis*. In animals, no FEMU2 homolog was found (Fig. 4B). Given that FEMU2 contains three copies of C2H2 ZF domains, the DNA sequences encoding for the three and two copies of C2H2 ZF domains were amplified by PCR with an upper primer, 5′-ATAAGAATTCCCGCTGTGCAAGTTCTGC-3′, and downstream primers 5′-AATCGTCGACGTGCTCTGCCGCGAAGTG-3′ and 5′-AATCGTCGACGGGGTGCGGGCAGGGGTG-3′ (Fig. 5A). Both amplified fragments were digested by *EcoR*I/*Sal*I and inserted into pGEX-6P-1 to construct the prokaryotic expression vectors pGEX3C2H2 and pGEX2C2H2, respectively. These two vectors were then transformed to *Escherichia coli* BL21 to express a 35.4 kD glutathione S-transferase (GST)-3C2H2 fusion protein and a 32.5 kD GST-2C2H2 fusion protein (Fig. 5B). PCR-mediated random binding-site selection revealed 57 clones with random DNA sequences binding to GST-3C2H2 fusion protein. Among these, 37 were observed as effective binding sequences after the repetitive sequences were removed. Among these 37 effective sequences, 20 contained a TGGT core sequence, accounting for 65% of the total sequences; 13 contained a TGGG core sequence, accounting for 35% of the total sequences; and 30 contained a TGGG/T core sequence, accounting for 81% of the total sequences. However, no DNA sequence binding to GST-2C2H2 was identified. As long oligonucleotides have more binding specificities to proteins than short ones, the probability of each base presenting in the border of TGGG/T core sequence of was analyzed. A total of 38 core sequences of 30 sequencing fragments were found, with some sequences containing more than one core sequence. Thus, (G/T)TTGG(G/T)(G/T)T was speculated as the FEMU2 3C2H2 ZF binding sequence (Fig. 6B).

### TTGGGT can bind to the FEMU2 3C2H2 ZF domain

**Figure 7:**
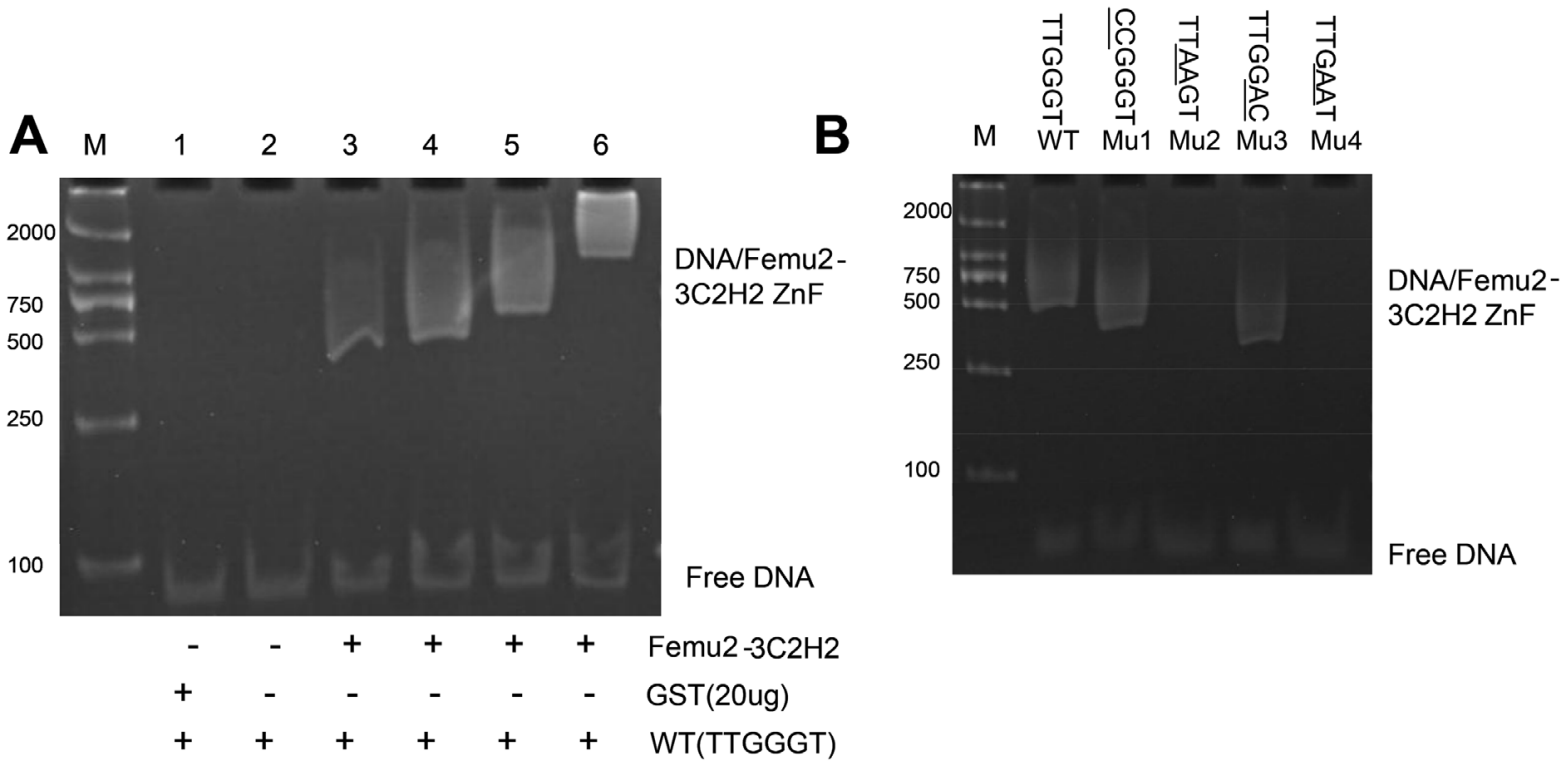
Binding assay of the core sequence TTGGGT oligonucleotide tetramer and its mutant sequence with FEMU2-3C2H2 ZF domain by EMSA. M, DL2000 molecular standard; 1∼6: specific competitive reaction. The amount of FEMU2-3C2H2 ZF is 0 µg (lanes 1 and 2), 10 µg (lane 3), 20 µg (lane 4), 30 µg (lane 5), and 40 µg (lane 6). WT, TTGGGT oligonucleotide tetramer; Mu1, *CC*GGGT oligonucleotide tetramer; Mu2, TT*AA*GT oligonucleotide tetramer; Mu3, TTGG*AC* oligonucleotide tetramer; Mu4, TT*GAA*T oligonucleotide tetramer.

To identify the binding of TTGGGT to the 3C2H2 ZF domain of FEMU2, five complementary oligonucleotide tetramers were synthesized and annealed to create double-stranded DNA. The resulting DNA was mixed with 3C2H2 ZF domain and loaded on a native PAGE. The results showed that TTGGGT (WT), Mu1, and Mu3 tetramers were bound to the Femu2-3C2H2 ZF (Fig. 7A), but Mu2 and Mu4 tetramers were not. The sequences of WT, Mu1, and Mu3 were the tetramers of TTGGGT, *CC*GGGT, and TTGG*AC*, respectively. For Mu2 and Mu4, the tetramers were TT*AA*GT and TT*GAA*T, respectively. This result indicated that G located on the third and fourth bases of the TTGGGT core sequence was significant in the binding of DNA to bind to the Femu2 3C2H2 ZF domain. Mutations of these two sites prevented TTGGGT from binding to the 3C2H2 ZF domain (Fig. 7B).

### FEMU2 positively regulates FOX1

**Figure 8:**
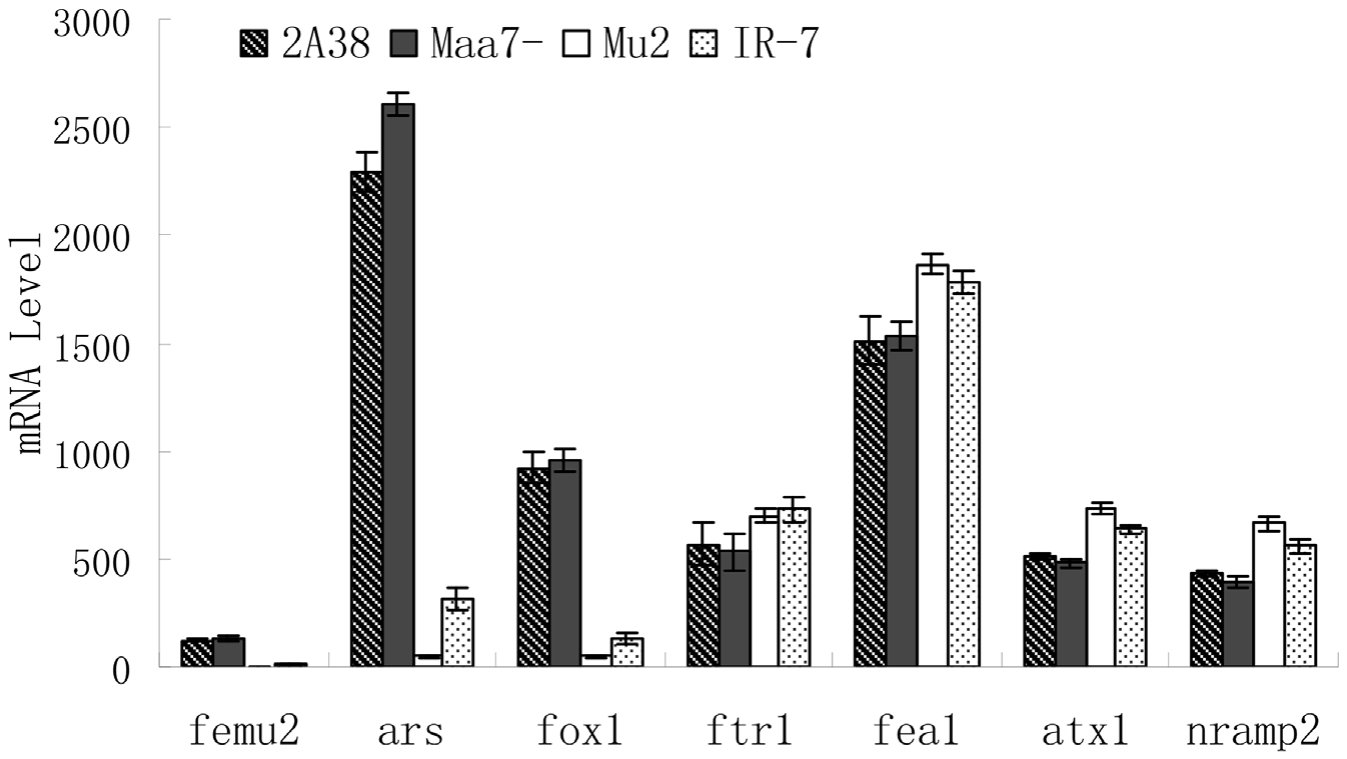
Comparison of the mRNA abundance of Fe-regulated genes. Strains IR7, mu2, 2A38, and Maa7- were grown for 4 days in Fe-deficient TAP medium. 2A38, *C. reinhardtii*. 2A38, CC425 with *fox1* promoter::*ars* expression cassette inserted in genome; Maa7-, transgenic algae with pMaa7IR/XIR transformed to *C. reinhardtii* 2A38; mu2, mutant with the *femu2* gene deleted from the genome of *C. reinhardtii* 2A38; IR7, pMaa7IR/Femu2IR transgenic algae.

As the chimeric gene *fox1* promoter::*ars* contains the −103 to +65 *fox1* promoter sequence and the *femu2* knockout and knockdown mutants showed decrease expression of ARS, we speculated that the *femu2* mutation may also affect the expression of endogenous *fox1* gene, which is controlled by the same promoter. IR7 and mu2 transgenic strains, *C. reinhardtii* 2A38 parent strain, and strains transformed with pMaa7IR/XIR were grown for 4 d in an Fe-deficient medium (0 µM Fe). The mRNA of each strain was extracted, and the corresponding *fox1* expression was analyzed. As shown in Fig. 8, *ars* transcript (Ct) abundance in mu2 and in IR7 decreased by 98.2% and 88.1% compared with that in the pMaa7IR/XIR control, respectively. Simultaneously, *fox1* Ct abundance in mu2 and in IR7 decreased by 94.7% and by 86.2%, respectively. These findings indicated that *femu2* knockout or silencing influenced *fox1* endogenous gene transcription. As *fox1* promoter::*ars* and *fox1* promoter::*fox1* contain the −103 to +65 *fox1* promoter sequence, FEMU2 may have direct or indirect interaction with this sequence, thus regulating *fox1* promoter::*ars* and *fox1* promoter::*fox1* expressions. Furthermore, the mRNA abundance of several known Fe-regulated genes was determined (Fig. 8). *ftr1* of mu2 and IR7 increased by 30.1% and 33.6% compared with that of the pMaa7IR/XIR transgenic strain; those of *FEA1* increased by 21.3% and 13.7%; those of *ATX1* increased by 47.1% and 28.0%; and those of *NRAMP2* increased by 70.2% and 44.5%, respectively.

### Subcellular localization of FEMU2

**Figure 9:**
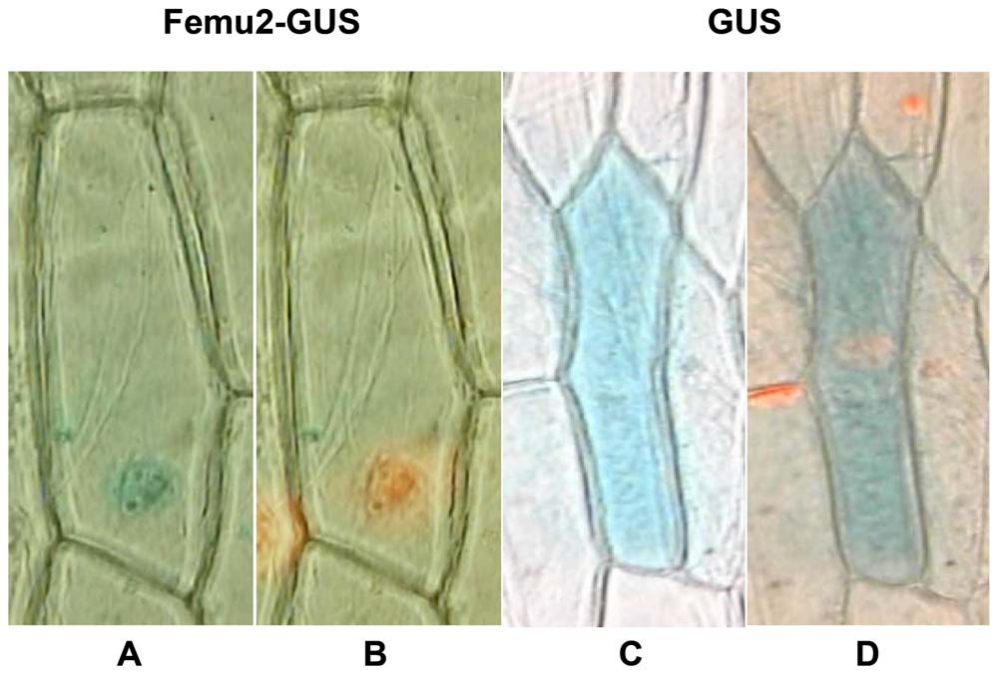
Subcellular localization of FEMU2. FEMU2-GUS: DNA sequence from 1593 to 2901 of FEMU2 fusion with GUS; GUS: GUS expression only. Substrate X-glucuronide was added to reveal the gene expression part (blue) (A, C); Propidium iodide was added to reveal parts of the nucleus (orange) (B, D).

FEMU2 was predicted to be localized in the nucleus by both Euk-mPLoc 2.0 and NLStradamus. NLStradamus predicted two NSLs in FEMU2; one was located at 612 to 619, with the sequence KGKGRAKV, and the other was located at 904 to 908, with the sequence KGKKG. The 1593 to 2901 *femu2* DNA fragment (amino acids 531 to 967) contained two NSLs fused with the β-glucuronidase (GUS) gene, followed by bombardment into onion epidermal cells. The results presented in Fig. 9 indicated that FEMU2 was localized in the nucleus, consistent with the predictions, and indicates that FEMU2 is involved in the regulation of Fe-related genes in the nucleus.

## Discussion

The molecular regulation of Fe metabolism has been a significant topic for a long time. However, only a few genes that contain Fe response elements and are involved in Fe regulation in plants and photosynthetic eukaryotes have been reported thus far [26]–[32]. Given the similarity between the Fe absorption mechanism of *Chlamydomonas* and that of plant root cells of higher plants (dicots and non-graminaceous monocots), i.e., strategy I, studies on Fe regulation in *Chlamydomonas* are particularly useful in investigating strategy I in plants [33]–[36]. Two Fe response elements in the Fe responsive gene promoters, namely, type I FeRE in *fox1* and type II FeRE in *atx1, ftr1*, and *fea1* have been demonstrated to be responsible for of Fe-deficiency-induced gene expression. In the current study, approximately 50,000 antibiotic-resistant transformants were obtained by transforming pSP124S to *C. reinhardtii* 2A38. The Fe deficiency responses of these mutants were then screened by phenotype analysis, which revealed that 68 transformants were valid mutants. A total of 23 candidate genes among these mutants were determined by conducting TAIL-PCR and by sequence analysis based on JGI *Chlamydomonas* genomic database. In mutant mu2, the identified defect gene *femu2* encodes a protein containing three copies of typical C2H2 ZF domains as predicted by Pfam and SMART (Fig. 4A). The results of cluster analysis showed that FEMU2 was closely related to its homologs in microalgae, fungi, and plants, but no homolog was found in animals.

Analysis of the C2H2 ZF domain-binding sequence indicated that the consensus sequence was (G/T)TTGG(G/T)(G/T)T (Fig. 6). Electrophoretic mobility shift assay (EMSA) was used to detect the binding of the WT core sequence of the TTGGGT tetramer and mutated sequences of CCGGGT, TTAAGT, TTGGAC, and TTGAAT tetramers to C2H2 ZF domains. The results showed that sequences other than TTAAGT and TTGAAT tetramers were bound to C2H2 ZF domains. In our previous study, two separate Fe-responsive elements were identified from the *fox1* promoter, FeRE1 (CACACG), and FeRE2 (CACGCG) between positions −87 and −82 and between positions −65 and 60, respectively, and were both necessary for *FOX-* induced expression under Fe-deficient conditions [25]. Does FEMU2 bind to *fox1* promoter through its C2H2 domain? From the sequence of *fox1* promoter -103 to +65, the consensus sequence (G/T)TTGG(G/T)(G/T)T was not found, which implies that FEMU2 could indirectly regulate the *fox1*–*ars* chimeric gene.

Interestingly, results obtained from the RNAi of *femu2* gene showed that *femu2* mRNA expression levels and ARS activities of the transformants decreased by 91.7% to 94.5% (Figs. 3, 2B, and 2C). These results indicate that FEMU2 regulated the *fox1* promoter of *fox1* promoter::*ars* chimeric gene in the transformants. Moreover, RNAi suppressed the expression of *femu2* gene, resulting in a decrease in ARS activity. *ARS*, *femu2*, and the Fe-regulated gene *fox1* mRNA levels in mu2 and IR7 also decreased compared to those of *C. reinhardtii* 2A38. Another interesting finding was that *atx1*, *ftr1*, *fea1*, and *nramp2* mRNA levels increased in the *femu2* gene knockout and knockdown strains compared to those of the control (Fig. 8). One possible explanation is that Fe regulation is promoted to higher levels due to decreased level of *fox1*, which directly affects Fe levels in the cell and leads to a general induction of genes involved in Fe reponses. FEMU2 protein could have also negatively regulated type II FeRE genes, such as *atx1*, *ftr1*, *fea1*, and *nramp2*. These results are similar to that of C2H2 domain-containing protein BTEB3, which binds the SM22*α* promoter in the TGGG core sequence [37]. Furthermore, BTEB3 can both activate and inhibit gene expression. For example, Martin et al. and Song et al. demonstrated that BTEB3 could activate a number of promoters [38], [39]. Kaczynski et al. showed that BTEB3 could inhibit gene activity by binding to the transcription co-repressor mSin3A [40]. Studies on BTEB1, another member of the BTEB subfamily of transcription factors, have suggested that BTEB1 functions as an activator of promoters with multimerized binding sites but as an inhibitor of promoters with single binding sites [41]. In addition, transcription factors with dual actions that are either activators or inhibitors include other ZF transcription factors. For example, yin yang 1 (YY1) activates certain promoters (e.g., c-Myc) but inhibits other promoters (e.g., SM*α*-actin and SM22*α* [42]). The ability of YY1 to act as an activator or inhibitor depends on the phase and orientation of specific YY1-binding site [43], [44].

Thus, our further research will focus on the interactions of FEMU2 with *atx1*, *ftr1*, *fea1*, and *nramp2* promoters. Chromatin co-precipitation technology will be used to study the binding between FEMU2 and DNA in vivo. Moreover, yeast two hybrid will be used to study the relationship between FEMU2 and proteins that directly interact with FeRE1 and FeRE2 of *fox1*. These results are considered useful references for studying Fe response and Fe uptake mechanism in plants, animals, and yeasts.

## Materials and Methods

### Bioinformatics

Information on the orthologous genes involved was retrieved from NCBI database using BLASTp and BLASTn of *femu2* (Cre12.g528400). The functional motifs of FEMU2 were predicted using Pfam (http://pfam.sanger.ac.uk/search) and SMART (http://smart.embl-heidelberg.de/). The subcellular localization of proteins was predicted using Euk-mPLoc 2.0 [45], [46] and NLStradamus. Sequence alignment of *femu2* and orthologous gene was conducted using ClustalW [47]. The phylogenetic tree was assembled using MEGA version 4.1 [48].

### Strains, culture conditions, chlorophyll contents, and Fv/Fm detections

*C. reinhardtii* 2A38, a transgenic strain with an integrated *fox1*-*ars* chimeric gene in *C. reinhardtii* CC425 genome [25], was used as the recipient strain in generating all the mutants. The mutants were grown in TAP liquid medium supplemented with 250 µg/mL arginine [49], [50]. The transformants were grown in either +Fe (18 µM Fe) or –Fe (0 µM Fe) TAP medium. Liquid cultures were grown at 25°C with agitation at 250 rpm under 150 µmol⋅m^−2^⋅s^−1^ of continuous light. The strains on TAP agar plates were incubated at 22°C under a 100 µmol⋅m^−2^⋅s^−1^ light intensity. To detect chlorophyll fluorescence, cells in the exponential phase were collected by centrifugation at 3,000×*g* for 5 min, and the resulting pellets were resuspended in 80% acetone and incubated in the dark for several hours until the cells turned gray. Chlorophyll fluorescence was detected at OD_663_, OD_646_, and OD_750_ using a 752N ultraviolet (UV)-visible spectrophotometer (Shanghai Precision & Scientific Instrument Co., Ltd. China) [51], [52]. Chlorophyll concentration was calculated using the following equation: Chl a (µg/mL)  = 12.21 (OD_663_ – OD_750_) – 2.81(OD_646_ – OD_750_); Chl b (µg/mL)  = 20.13 (OD_646_ – OD_750_) – 5.03(OD_663_ – OD_750_). Chlorophyll fluorescence parameters Fv/Fm were measured with a modulated fluorometer (Mini PAM Photosynthesis Yield Analyzer, Walz, Effeltrich, Germany) according to the manufacturer's instructions. Briefly, algal strains were grown for 6 d in TAP medium without Fe, Cu, Mn, or Zn and then cultured for 20 min under dark conditions. The special fiber optics was cast in the media, and data were collected in the dark. The experiment was repeated three times. Independent sample t-test was used for data analysis.

### Generation and screening of mutants

*Chlamydomonas* cells were subjected to insertion mutagenesis according to the transformation method proposed by Kindle et al. [53]. *Kpn*I-linearized pSP124S plasmid was used in the transformation [54]. Approximately 2 µg of plasmid DNA was used for pSP124S transformation. The cells were then allowed to recover overnight in 10 mL TAP under dark conditions at 25°C with shaking at 150 rpm. The cells were collected by centrifugation at 1,500×*g* for 3 min, resuspended in 300 mL TAP, and plated onto TAP agar plates containing 10 µg/mL of zeocin (Invitrogen). After 7 d, the colonies formed were transferred onto +/– Fe TAP agar plates in duplicates. ARS activity was determined as described by Davies et al. [55]. 5XSO4 (10 mM; Sigma Chemical Co.) was added to plates with –Fe TAP solid medium. The plates were etched before clones were inoculated. After 1 d, transformants that did not express ARS activity and those without blue halo around their colonies were chosen for further investigations.

### Genomic DNA preparation and gel blot analysis

*Chlamydomonas* cells in the mid-log phase were collected for genomic DNA extraction according to the method proposed by Deng and Erikson with modifications [25]. Algae cells were pelleted and centrifuged at 12,000 rpm for 30 s. The supernatant was aspirated, and the cells were resuspended in H_2_O. SDS-Buffer (2% SDS, 400 mM NaCl, 40 mM Na_2_EDTA, 100 mM Tris-HCl, pH 8.0), RNase (20 µg/µL), and acid-washed glass beads (Sigma, St. Louis, MO, USA) were added to the cells. The resulting solution was mixed in a vortex and kept at room temperature for 15 min. After a series of phenol–chloroform extractions, DNA was precipitated with two volumes of absolute ethanol and then washed with 70% ethanol. The pellet was air dried and dissolved in TE buffer (10 mM tris-HCl, 1 mM EDTA, pH 8.0). Genomic DNA concentration was determined using a spectrophotometer, and DNA integrity was verified using agarose gel electrophoresis. For DNA gel blot analysis of the insertion mutants, genomic DNA was digested with *EcoR*I or *Hind*III. The probe used was a P^32^-labeled 120 bp *ble* gene coding sequence.

### TAIL-PCR

Genomic DNA adjacent to the insertion site of the transforming DNA was amplified by TAIL-PCR [56]. The method used was optimized for *Chlamydomonas*. The specific primers for the primary, secondary, and tertiary reactions were ChlamyTail1 (5′-CCGAGGAGCAGGACTAACCG-3′), ChlamyTail2 (5′-GATCCCCGCTCCGTGTAAATG-3′), and ChlamyTail3 (5′-ACGGCGGTGGATGGAAGATA-3′), respectively. Six arbitrary and degenerate primers were amplified, namely, AD1 (5′-NTCGA(G/C)T(A/T)T(G/C)G(A/T)GTT-3′), AD2 (5′-NGTCGA(G/C)(A/T)GANA(A/T)GAA-3′), AD3 (5′-(A/T)GTGNAG(A/T)ANCANAGA-3′), AD4 (5′-AG(A/T)GNAG(A/T)ANCA(A/T)AGG-3′), AD5 (5′-NTCGWGWTSCNAGC-3′), and AD6 (5′- WGNTCWGNCANGCG-3′) [57]. AD1, AD2, AD3, and AD4 primers were designed by Liu [56], and AD5 and AD6 were designed by Dent [58].

AD1 and AD2 successfully amplified the flanking regions of most of the samples. By contrast, AD3, AD4, AD5, and AD6 only resulted in fragments in 30% of the samples tested. Therefore, AD1 and AD2 were selected as degenerate primers for all future reactions. Primary TAIL-PCR reactions contained PCR buffer (500 mM KCl, 100 mM Tris-HCl, 15 mM MgCl_2_, pH 8.3), 10 mM of each dNTP, 10 pmol ChlamyTail 1, 50 pmol RMD227, and 2.5 U Taq polymerase. The cycling parameters for the TAIL-PCR reactions are presented in Table 2.

**Table 2:** Primers used for real-time RT-PCR and amplifying the genes around the insertion site of mu2.

Gene name	Primer sequence
18S gene	Forward primer: 5'-TCAACTTTCGATGGTAGGATAGTG-3';
	Reverse primer: 5'-CCGTGTCAGGATTGGGTAATTT-3'
*femu2*	Forward primer: 5′-CCTTCAACGCAGAGGACTTC-3′;
	Reverse primer: 5′-TGAATGCAGCCATACTGGAA-3′
*ars*	Forward primer: 5′-ATGGGTGCCCTCGCGGTGTTC-3′;
	Reverse primer: 5′-GTAGCGGATGTACTTGTGCAG-3′
*fox1*	Forward primer: 5′-GACGTGGAGGCCCAGAAG-3′;
	Reverse primer: 5′-CGCGACGAAGTAGGTGTTG-3′
*ftr1*	Forward primer: 5′-TCTTTCGGGAGACCATTGAG-3′;
	Reverse primer: 5′-GAAGCATAGCAAAGCCAAGG-3′
*fea1*	Forward primer: 5′-CTCAAGTACCACCTGCACGA-3′;
	Reverse primer: 5′-ACATAGCTCTTGCCGAGGAA-3′
*atx1*	Forward primer: 5′-AGCTCGTGTCCTCGTAAAGC-3′;
	Reverse primer: 5′-CTGCAACAGGTTCCGTGTAA-3′
*nramp2*	Forward primer: 5′-CTGTCGCAGGTGATCCTGT-3′;
	Reverse primer: 5′-TTTGCACCACCAGGTTAATG-3′;
192563	Forward primer: 5′-CGGGCTAGGAAATACAACCA-3′;
	Reverse primer: 5′-CCCTCTGTAACATCCGCATT-3′;
81238	Forward primer: 5′-GTCGCCTTTATCCCCTTCAT-3′;
	Reverse primer: 5′-AACAAGCGATCTCCTGCACT-3′;
176119	Forward primer: 5′-GCCCTCAGCCTAACAAACTG-3′;
	Reverse primer: 5′-GACACCAGGGCAGCTTCTAC-3′;
150664	Forward primer: 5′-GCGGAGGAGTACCACGACTA-3′;
	Reverse primer: 5′-GCATGTACAGCACCCACAAC-3′;
192574	Forward primer: 5′-AGAGCCCATGTCGTGTTACC-3′;
	Reverse primer: 5′-AACCCCCGCTATTTGACTCT-3′;
138808	Forward primer: 5′-ATCAGCGGTGGCTTCTTCTA-3′;
	Reverse primer: 5′-TTGTTCTTGTCACGGCTGAG-3′;

Primary reaction mixtures were diluted by 40-folds and used for secondary TAIL-PCR reactions, which have similar components and concentrations to those of the primary reaction, except for the specific primer, which was replaced with ChlamyTail 2. For less astringent tertiary reaction, the secondary reaction was diluted by 20-folds. The components for the tertiary reaction were similar to those of the primary reaction, with ChlamyTail 2 as the specific primer. The amplified products from both reactions were analyzed by agarose gel electrophoresis. The purified PCR products were cloned in pMD18-T vector (Takara, China) and then sequenced.

### *In vitro* expression of FEMU2 C2H2 ZF domain

The DNA fragment of *femu2* C2H2 ZF domain was amplified using the following primers: F-C2H2 5 ′-ATAAGAATTCCCGCTGTGCAAGTTCTGC-3′, R-C2H2 5′-AATCGTCGACGTGCTCTGCCGCGAAGTG-3′, F-C2H2P 5′-ATAAGGATCCTGCAAGTTCTGCCGGCAG-3′, and R-C2H2P 5′-AATCGAATTCGGGGTGCGGGCAGGGGTG-3′. The purified fragment was inserted into pGEX-6P-1 and then transformed in *E. coli* DH5α. The plasmids of the aforementioned positive clone were extracted and transformed by *E. coli* BL21. Afterwards, isopropyl- β-D-1-thiogalactopyranoside (IPTG) induction was conducted. The bacteria were collected by centrifugation, and the pellets were resuspended in 1× SDS loading buffer. Sample proteins were resolved by 15% SDS-PAGE and stained by Coomassie brilliant blue. These proteins were purified by affinity chromatography as described in the Purification Procedure B of GST Sefinose Resin instructions (Sangon Biotech, Shanghai, China). The samples were passed through a membrane filter with 0.45 µm pore diameter. The eluted proteins were enriched by centrifugation at 5,000×g for 30 min in PALL ultrafiltration tubes, and their concentrations were identified.

### PCR-mediated random binding site selection [59]

A 60-base oligonucleotide called 60N18, 5′-ATTCAGATCTTAAACACAGGA…N18…GTGATGCTCGGTACCCTAAAG-3′, was synthesized and used as PCR template with primer N18P1 [5′-CGCGAATTCATTCAGATCTTAAACA-3′ (*EcoR*I)], and primer N18P2 [5′-TTCGTCGACCTTTAGGGTACCGAGC-3′(*SalI*)]. The amplified fragments were purified, recovered, and incubated in a GST-C2H2 ZF column at 4°C for 30 min. The column was washed thrice with DNA-binding buffer and then with elution buffer. The eluent was boiled for 10 min and centrifuged to precipitate the DNA in the supernatant with an equal volume of ethanol. The obtained DNA was used to amplify the fragments by PCR. After three to five cycles of the aforementioned steps, the amplified DNA was digested with *EcoRI* and *Sal* restriction enzymes and subcloned in a pGEX-6P-1 vector. The DNA sequences of the inserts in individual clones were then determined.

### FEMU2 C2H2 ZF domain DNA-binding assay

An EMSA kit (E33075; Invitrogen) was used to determine the binding of the *Femu2* C2H2 ZF domain to DNA. Five pairs of 24-bp single-stranded oligonucleotides were synthesized (Table 3), with WT, Mu1, Mu2, and Mu3 as the mutated TTGGGT, CCGGGT, TTAAGT, TTGGAC, and TTAAAT tetramers, respectively. The five pairs of single-stranded oligonucleotides, namely, WT-p1/WT-p2, Mu1-p1/Mu1-p2, Mu2-p1/Mu2-p2, Mu3 -p1/Mu3-p2, and Mu4 -p1/Mu4-p2, were mixed equally. The mixtures were denatured at 95°C for 5 min and then cooled to room temperature to obtain double-stranded DNAs. Double-stranded WT was used as competitor to various amounts of FEMU2-C2H2 protein with GST as the control sample. Double-stranded WT was mixed with 10, 20, 30, and 40 µg of FEMU2-C2H2 protein. Approximately 1/10 volume of DNA-binding buffer was added, and the resulting mixture was incubated at 22°C for 20 min for the binding reaction. Results were determined by 8% native PAGE [60]. After electrophoresis, the gel was removed and stained with 1× SYBR Green EMSA stain/TBE buffer at room temperature for 30 min. Sample binding was detected by UV transillumination at 300 nm.

**Table 3:** The oligonucleotide sequence used in binding test of Femu2-3C2H2 Znf by EMSA.

Name		*Oligonucleotide sequence*
WT	WT-p1	5′-***TTGGGT*** TTGGGT TTGGGT TTGGGT-3′
	WT-p2	5′-ACCCAA ACCCAA ACCCAA ACCCAA-3′
Mu1	Mu1 -p1	5′-***CC***GGGT ***CC***GGGT ***CC***GGGT ***CC***GGGT-3′
	Mu1-p2	5′-ACCCGG ACCCGG ACCCGG ACCCGG-3′
Mu2	Mu2-p1	5′-TT***AA***GT TT***AA***GT TT***AA***GT TT***AA***GT-3′
	Mu2-p2	5′-ACTTAA ACTTAA ACTTAA ACTTAA-3′
Mu3	Mu3-p1	5′-TTGG***AC*** TTGG***AC*** TTGG***AC*** TTGG***AC***-3′
	Mu3-p2	5′-GTCCAA GTCCAA GTCCAA GTCCAA-3′
Mu4	Mu4-p1	5′-TT***GAA***T TT*G* ***AA***T TT***GAA***T TT***GAA***T-3′
	Mu4-p2	5′- ATTCAA ATTCAA ATTCAA ATTCAA-3′

### RNA extraction

Total RNA was extracted using TRIzol reagent (Sangon Biotech, Shanghai, China) according to the procedure described by Deng et al. with modifications [61]. Algae cells were collected by centrifugation at 10,000×*g* for 1 min. After a series of phenol–chloroform extractions, nucleic acid was precipitated with two volumes of absolute ethanol and then washed with 75% ethanol. The resulting pellet was air dried and dissolved in RNase-free water. RNA concentration was determined by spectrophotometry, and RNA integrity was examined by agarose gel electrophoresis.

### Construction of RNAi vectors against *femu2* gene

A fragment of *C. reinhardtii* 18S gene was amplified using 5′-CGAACTTCTGCGAAAGCAT-3′ and 5′-TCAGCCTTGCGACCATACT-3′ primers. This fragment was inserted in pMD18-T to obtain pMD18T-18S and construct an RNAi vector against the *femu2* gene. The *femu2* fragment and its reverse complementary sequences were amplified by PCR using *C. reinhardtii* cDNA as template. The following primers were used: 5′-GCCGAAGCTTCCTTCAACGCAGAGGACTTC-3′, 5′-GCCGTCTAGATGAATGCAGCCATACTGGAA-3′, 5′-GCCGGTCGACCCTTCAACGCAGAGGACTTC-3′, and 5′-GCCGTCTAGATGAATGCAGCCATACTGGAA-3′. The fragments were then digested with *Kpn*I/*Bam*HI and *Hin*dIII/*Sal*I and subsequently inserted into the corresponding cloning sites of pMD18T-18S to obtain pMD18-Femu2F-18S-Femu2R, which contains the inverted repeat sequence of *femu2* (*femu2* IR). pMD18-Femu2F-18S-Femu2R was digested with *Kpn*I and *Hin*dIII to obtain *femu2* IR. *Femu2* IR was inserted as a blunt-end fragment in *Eco*RI, which was then digested with pMaa7/XIR to obtain pMaa7IR/*Femu2* IR.

### Transformation of *Chlamydomonas*

*C. reinhardtii* strain CC425 was transformed as described by Kindle [53]. *C. reinhardtii* cells were grown to a cell density of 1×10^6^ cells/mL to 2×10^6^ cells/mL in TAP medium. The cells were collected by centrifugation, washed twice, and resuspended in TAP medium to a cell density of approximately 1×10^8^ cells/mL. Plasmid DNA was introduced to the cells using the glass bead procedure. In each case, 2 µg of plasmid DNA was included in a mixture containing 400 µL cells, 100 µL 20% polyethylene glycol, and 300 mg sterile glass beads. The reaction mixture was mixed for 15 s on a bench-top vortex. The cells were allowed to recover for 1 d and then plated on a selective medium to induce RNAi or gene expression. pMaa7IR/*Femu2*IR transformants were selected on a TAP medium containing 1.5 mM L-tryptophan, 5 µg/mL paromomycin, and 5 µM 5-fluoroindole. Transformants were selected on a TAP medium containing 50 µg/mL kanamycin. The plates were incubated under dim light (approximately 50 µmol⋅m^−2^⋅s^−1^ of photosynthetically active radiation). Isolated transgenic strains were kept under a constant selected pressure.

### ARS activity assay

ARS activity was determined as described by Davies and Grossman [62]. XSO_4_ (10 mM) was added to plates with –Fe TAP solid medium and scribed before clones were inoculated. After 1 d, transformants that expressed ARS activity were identified by blue halos around their colonies. To analyze ARS activity quantitatively, the cells were initially collected by centrifugation. The supernatant was added with 0.1 M glycine–NaOH at pH 9.0, 10 mM imidazole, and 4.5 mM *p*-nitrophenyl sulfate. The reaction mixture was incubated at 27°C for 30 min. The reaction was terminated by adding 0.25 M NaOH, and its absorbance at 410 nm was determined. A standard curve of *p*-nitrophenol (Sigma Chemical Co.) was obtained using 0.2 M NaOH [63].

### Real-time PCR

The transformants used for real-time PCR analysis were cultured in –Fe (0 µm) and + Fe (18 µm) TAP liquid media to a cell density of 2×10^6^ cells/mL to 5×10^6^ cells/mL. RNA was extracted using TRIzol reagent (Sangon Biotech, Shanghai, China). Single-strand cDNA was obtained using 100 ng of the RNA sample and a random primer at 65°C for 5 min, 25°C for 5 min, and 42°C for 50 min in a Bio-Rad iScript select cDNA synthesis kit. Real-time PCR was conducted in a BioRad iCycler iQ real-time PCR detection system by using SYBR Green as the fluorescent dye. Each reaction was conducted in a final volume of 25 µL with the following components: 0.2 pmol of each primer, 1 µL of cDNA, and 12.5 µL of SYBR Green Mix (Invitrogen SYBR Greener QPCR). Water was used to adjust the volume to 25 µL. The iCycler run protocol was performed as follows: denaturation at 95°C for 5 min, 40 cycles of denaturation at 95°C for 30 s, annealing at 54°C for 30 s, and amplification at 72°C for 15 s. The specificity of PCR amplification was verified using a melting curve program (from 55°C to 100°C at a heating rate of 0.5°C/s). 18S rRNA was used as the control sample with primers 18SrRNAF (5′-TCAACTTTCGATGGTAGGATAGTG-3′) and 18SrRNAR (5′-CCGTGTCAGGATTGGGTAATTT-3′). The expression of the control gene was determined and found to be constant under all of the conditions used in this study. Primers (5′-CCTTCAACGCAGAGGACTTC -3′) and (5′- TGAATGCAGCCATACTGGAA -3′), specific for the *femu2* gene, were used to evaluate the mRNA level. The amplification rate of each transcript (Ct) was calculated using the PCR base line subtracted method, which was conducted in iCycler software at a constant fluorescence level. Cts were determined with three repeats. The relative fold differences were calculated on the basis of the relative quantification analytical method (2^-ΔΔCT^) by using 18S rRNA amplification as an internal standard [64].

### Transient expression of FEMU2 fragments in onion epidermal cells

Fusion proteins between the fragments of FEMU2 and GUS protein were constructed to examine the intracellular localization of these proteins in *Chlamydomonas* under the control of cauliflower mosaic virus 35S promoter. The fragment from 1593 to 2901 of *Femu2* containing two of the nuclear localization sites predicted by NLStradamus was amplified by PCR using ACTTCTAGA **ATG**CCGCCGGCGGTCTCGCCGT and TGCGGATCCCTCTTCGCCCCACGCGTCGT primers. The obtained fragment was modified by adding a translation start codon in its 5′end and removing the stop codon in its 3′ end. This fragment was then inserted into pBI121 plasmid in the *Xba*I/*BamHI* site fusing with the GUS gene. The epidermal cells of onion bulbs were transformed through microprojectile bombardment with DNA-coated tungsten particles [65], [66]. After bombardment, the epidermal peels were incubated for 24 h to 30 h at 23°C under a constant light intensity of 50 µmol m^−2^ s^−1^ of photosynthetically active radiation. The cells were examined under microscopy to determine GUS activity distribution.
